# Ultrasensitive Ochratoxin A Detection in Cereal Products Using a Fluorescent Aptasensor Based on RecJ_f_ Exonuclease-Assisted Target Recycling

**DOI:** 10.3390/foods13040595

**Published:** 2024-02-16

**Authors:** Yanxuan Li, Furong Shao, Jin Wu, Mingzhu Liu, Gaofang Cao, Zunquan Zhao, Jialei Bai, Zhixian Gao

**Affiliations:** 1 Tianjin Key Laboratory of Risk Assessment and Control Technology for Environment and Food Safety, Tianjin Institute of Environmental and Operational Medicine, Tianjin 300050, China; liyanxuan2020@163.com (Y.L.); furong500823@163.com (F.S.); wujinlch@163.com (J.W.); lmzazhu@163.com (M.L.); baijialeitj@163.com (J.B.); gaozhx@163.com (Z.G.); 2 Department of Public Health and Management, Binzhou Medical University, Yantai 264003, China; caogaofang2003@126.com

**Keywords:** fluorescence sensor, aptasensor, RecJ_f_ exonuclease, target recycling, ochratoxin A

## Abstract

Ochratoxin A (OTA) is a mycotoxin widely found in foodstuffs such as cereal grains. It greatly threatens human health owing to its strong toxicity and high stability. Aptasensors have emerged as promising tools for the analysis of small molecule contaminants. Nucleic-acid-based signal amplification enables detectable signals to be obtained from aptasensors. However, this strategy often requires the use of complex primers or multiple enzymes, entailing problems such as complex system instability. Herein, we propose a fluorescent aptasensor for the ultrasensitive detection of OTA in cereal products, with signal amplification through RecJ_f_ exonuclease-assisted target recycling. The aptamer/fluorescein-labeled complementary DNA (cDNA-FAM) duplex was effectively used as the target-recognition unit as well as the potential substrate for RecJ_f_ exonuclease cleavage. When the target invaded the aptamer-cDNA-FAM duplex to release cDNA-FAM, RecJ_f_ exonuclease could cleave the aptamer bonded with the target and release the target. Thus, the target-triggered cleavage cycling would continuously generate cDNA-FAM as a signaling group, specifically amplifying the response signal. The proposed exonuclease-assisted fluorescent aptasensor exhibited a good linear relationship with OTA concentration in the range from 1 pg/mL to 10 ng/mL with an ultralow limit of detection (6.2 ng/kg of cereal). The analytical method showed that recoveries of the cereal samples ranged from 83.7 to 109.3% with a repeatability relative standard deviation below 8%. Importantly, the proposed strategy is expected to become a common detection model because it can be adapted for other targets by replacing the aptamer. Thus, this model can guide the development of facile approaches for point-of-care testing applications.

## 1. Introduction

Mycotoxins are toxic metabolites of certain fungal species that grow on various foodstuffs and animal feeds [1]. Ochratoxin A (OTA) is a very widespread mycotoxin, mainly produced by *Penicillium* and *Aspergillus* species, which is often detected in agricultural products including cereal grains such as wheat and corn, plant-derived products [2], and animal-derived products [3]. OTA can withstand food processing because of its high chemical and thermal stability and is known to induce nephrotoxic, immunotoxic, possibly carcinogenic, and other detrimental effects [4,5]. Referring to European Union standards, the maximum permitted level of OTA in cereal raw materials was 5 μg/kg, while 3 μg/kg was set for cereal-based foods [6]. As OTA consumption poses a great threat to human and animal health, the determination and quantification of OTA levels in contaminated raw foods and feeds are of vital importance.

Conventional chromatographic methods, such as high-performance liquid chromatography (HPLC) and liquid chromatography−mass spectrometry, have generally been employed as reference techniques for OTA detection. However, they often require sophisticated instruments, time-consuming sample pretreatment, and skilled personnel. Antibody-based analytical methods, including enzyme-linked immunosorbent assay (ELISA) and lateral flow immunoassay, are alternatives to instrumental methods for OTA detection. Nevertheless, most of the traditional immunoanalysis methods suffer from several limitations owing to the intrinsic properties of antibodies, such as the long preparation period, strict storage conditions, and high cost. Given that aptamers have been considered good alternatives to antibodies, emerging aptamer-based methods have attracted increasing attention for the rapid and economical detection of OTA.

Aptamers, a class of functional oligonucleotides that bind to their targets with high affinity and specificity, are usually generated in vitro using the systematic evolution of ligands through exponential enrichment [7]. Compared to other analytic ligands, aptamers possess many compelling properties, such as small size, desirable chemical stability, facile synthesis and modification, and versatile structural design and engineering [8]. A series of strategies have been developed to convert target–aptamer binding signals into colorimetric [9], electrochemical [10], fluorescent [11], or other detectable signals [12,13]. Efficient signal amplification strategies are often required for sensitive or ultrasensitive fluorescence detection. Nucleic-acid-based signal amplification strategies are attractive options for the task because they capitalize on the intrinsic properties of aptamers. Specifically, isothermal amplification methods, such as loop-mediated isothermal amplification [14], rolling circular amplification [15], and strand-displacement amplification [16], have been widely applied in aptamer-based analyses. However, these approaches usually require the use of complex primers or multiple enzymes [14,15,16,17] and face issues such as complex system instability and non-specific amplification [18].

Exonuclease-assisted target recycling amplification is an alternative method that does not suffer from the drawbacks of nucleic acid amplification. Specifically, the RecJ_f_ exonuclease, as a single-stranded DNA (ssDNA)-specific exonuclease, can catalyze the removal of deoxy-nucleotide monophosphates from ssDNA in the 5′-to-3′ direction. Whereas other exonucleases usually act only on individual DNA strands [19,20], the RecJ_f_ exonuclease can also gradually hydrolyze the aptamer–target complex from the DNA strand and release the target for recycling [21,22,23]. Compared with nucleic acid amplification methods, RecJ_f_ exonuclease-induced target recycling can constantly amplify specific signals that are dependent on target aptamer recognition. A type of specific signal amplification can be achieved without complex primer design or the addition of multiple enzymes, thus avoiding the instability and non-specific amplification associated with complex system. RecJ_f_ exonuclease-induced signal amplification has been widely applied in a variety of electrochemical aptasensors [21,22,23], but it has seldom been used for fluorescence assays. Currently, fluorescence analysis is an accessible detection method that has reached a high level of commercialization. Herein, we introduce RecJ_f_ exonuclease-induced signal amplification enabling ultrasensitive fluorescence analysis in a universal aptamer-based magnetic separation system. Fluorescein-labeled complementary DNA (cDNA-FAM) was first hybridized with aptamers immobilized on magnetic beads, making the 5′ terminal of the aptamer hidden in the aptamer/cDNA-FAM duplex in the absence of targets. When the aptamer/cDNA-FAM duplex recognizes target OTA, cDNA-FAM is released into the supernatant, forming an OTA/aptamer complex on magnetic beads. At the same time, RecJ_f_ digests the ssDNA aptamer in the OTA/aptamer complex in the direction from 5′ to 3′, resulting in the release of the target OTA. Thus, the target OTA continuously exposes the 5′ terminal of the aptamer and triggers cleavage cycling to efficiently amplify cDNA-FAM signals in the supernatant. Through this mechanism, the aptasensor may be used to detect OTA with excellent sensitivity and specificity. Importantly, the proposed method exhibited good practical performance in OTA detection in cereal products, suggesting its practical applicability in food safety analysis.

## 2. Materials and Methods

### 2.1. Materials and Reagents

RecJ_f_ exonuclease was purchased from New England Biolabs (Beijing) Ltd. (Beijing, China). Streptavidin-coated magnetic beads (SMBs, 1 μm) were obtained from Beaver Biosciences Inc. (Suzhou, China). OTA, deoxynivalenol (DON), aflatoxin B1 (AFB1), ochratoxin B (OTB), and ochratoxin C (OTC) were purchased from Alta Scientific Ltd. (Tianjin, China). Methanol and acetonitrile (HPLC grade) were purchased from Sigma–Aldrich (USA). Tris(hydroxymethyl)aminomethane hydrochloride (Tris-Hydrochloride), sodium chloride (NaCl), and magnesium chloride (MgCl_2_) were obtained from Aladdin Ltd. (Shanghai, China). Fresh buffer Ⅰ (10 mM Tris-HCl, 50 mM NaCl, 10 mM MgCl_2_, pH 6.5) was prepared whenever required. A commercial ELISA Kit for OTA (limit of detection of 1.5 μg/kg) was purchased from Cusabio Biotech Ltd. (Wuhan, China). Milli-Q water (18.2 MΩ, deionized water) was used throughout the study.

All oligonucleotides, purified via HPLC, were supplied by Sangon Biotechnology Ltd. (Shanghai, China) and Genewiz Inc. (Suzhou, China). The DNA sequences are listed below.

Aptamer: 5′-AAAGATCGGGTGTGGGTGGCGTAAAGGGAGCATCGGACAAAAAA-Biotin-3′; cDNA-FAM: 5′-AAACCGATGCTCCCTTTACGCCACCCACACCCGATC-6-FAM-3′. The cDNA-FAM was complementary to the aptamer. 

### 2.2. Apparatus

Fluorescence analysis was performed using an F97pro fluorescence spectrophotometer (Shanghai Lengguang Technology Ltd., Shanghai, China) and a SpectraMax M5 microplate reader (Molecular Devices, San Jose, CA, USA). Scanning electron microscopy (SEM) images were recorded using a Quattro environmental high-resolution scanning electron microscope (Thermo Scientific, Waltham, MA, USA). Transmission electron microscopy (TEM) images were recorded using a Talos F200X transmission electron microscope (Thermo Scientific, USA). Confocal laser scanning microscopy (CLSM) was performed using an Eclipse Ti2-E confocal laser scanning microscope (Nikon, Tokyo, Japan). The circular dichroism spectrum was acquired on a Chirascan circular dichroism spectrometer (Applied Photophysics, Leatherhead, UK). Zeta potential values were recorded using a ZS90 Zetasizer Nano instrument (Malvern Panalytical, Malvern, UK). Polyacrylamide gel electrophoresis (PAGE) for DNA analysis was conducted on a Bio-RAD electrophoresis system, and the images were captured using an Amersham Imager 680 imaging system (GE Healthcare, Waukesha, WI, USA).

### 2.3. Fabrication of SMBs Modified with an Aptamer and cDNA-FAM (FAM-cDNA-Apt-SMBs)

A 40 μL aliquot of the suspension of commercial SMBs (10 mg/mL) was placed in the magnetic separator for 3 min before removing the supernatant. Then, the SMBs were washed with 100 μL buffer Ⅰ (10 mM Tris-HCl, 50 mM NaCl, 10 mM MgCl_2_, pH 6.5) three times and dispersed in 100 μL of buffer Ⅰ. Subsequently, the SMBs were mixed with 4 μL of the aptamer solution (100 μM) in ultrapure water and incubated for 30 min (25 °C, 300 rpm). The beads were washed with 100 μL of buffer Ⅰ three times to remove the excess aptamer strands. Then, the aptamer-modified beads (Apt-SMBs) were suspended in 100 μL of buffer Ⅰ. Next, 4 μL of a cDNA-FAM solution (100 μM) was added and incubated for 30 min (25 °C, 300 rpm). After the hybridization reaction, the modified SMBs (FAM-cDNA-Apt-SMBs) were washed with 100 μL of buffer Ⅰ five times. Finally, the prepared FAM-cDNA-Apt-SMBs were stored at 4 °C in 100 μL of buffer Ⅰ for future use.

### 2.4. Fluorescence Detection of OTA

Briefly, 20 μL of OTA standard solution of different concentrations and 1 μL of the RecJ_f_ exonuclease (30 U/μL) were added to a 20 μL aliquot of FAM-cDNA-Apt-SMB suspension and incubated at 37 °C for 75 min. The supernatant (10 μL) after magnetic separation was collected, heated at 65 °C for 20 min to inactivate the RecJ_f_ exonuclease, and diluted to 100 μL with buffer Ⅰ for fluorescence measurement. The fluorescence emission spectra of the solution were recorded on an F97pro fluorescence spectrophotometer at an excitation wavelength of 480 nm, with emission start at 508 nm, emission termination at 600 nm, an excitation slit of 20 nm, an emission slit of 10 nm, and high gain of 1000 V. In some experiments, fluorescence intensities were collected using a SpectraMax M5 microplate reader at 520 nm emission wavelength and 480 nm excitation wavelength. All measurements were repeated three times.

### 2.5. Pretreatment of Food Samples

Wheat and corn flours were acquired from a local supermarket in Tianjin. Briefly, 5 g NaCl was mixed with 25 g wheat or corn flour, extracted with 80% methanol to 100 mL, and sonicated for 10–20 min. The extracted supernatant was filtered through a qualitative filter paper. The filtered supernatant (10 mL) was transferred to a 50 mL volumetric flask, diluted with Milli-Q water to 50 mL, and thoroughly mixed. The crude filtrate was filtered using glass-fiber filter paper until the filtrate was clear for subsequent analysis. Cereal samples were spiked with various doses (0.2, 2.0, and 20.0 μg/kg) of OTA before extraction.

## 3. Results and Discussion

### 3.1. Sensing Principle of Fluorescence Assay

**Scheme 1:**
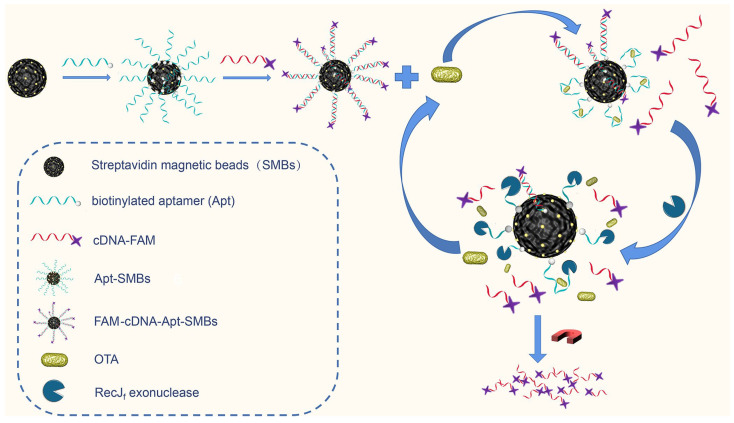
Schematic of the exonuclease-assisted aptamer sensing strategy for OTA detection.

The design of the proposed fluorescence assay is outlined in Scheme 1, which employs an aptamer-biosensing strategy using a fluorescent magnetic probe (FAM-cDNA-Apt-SMBs) and RecJ_f_ exonuclease-assisted target recycling amplification. For the self-assembly of the aptamer/cDNA-FAM duplex on the magnetic probe, the 3′ terminal-biotinylated aptamer was first immobilized on the surface of SMBs through streptavidin–biotin conjugation. Subsequently, 6-carboxyfluorescein-labeled complementary DNA (cDNA-FAM) was self-assembled to form the aptamer/cDNA-FAM duplex through DNA hybridization. In the absence of target molecules (OTA), the 5′ terminal of the aptamer, which is hidden in the aptamer/cDNA-FAM duplex, cannot be recognized by the RecJ_f_ exonuclease. When the target OTA is present, conformational switching of aptamers specific for OTA binding can lead to the formation of the OTA/aptamer complex as well as the release of cDNA-FAM from the aptamer/cDNA-FAM duplex. At the same time, the 5′ terminal of the aptamer in the OTA/aptamer complex is exposed to cleavage by the RecJ_f_ exonuclease, resulting in stepwise hydrolysis of the aptamer ssDNA in the 5′-to-3′ direction. Thus, the cleavage of the aptamer by the RecJ_f_ exonuclease is sufficient to release the target OTA from the OTA/aptamer complex, thereby enabling the reuse of the target to react with another aptamer/cDNA-FAM duplex. As a result, an increasing amount of cDNA-FAM is released from FAM-cDNA-Apt-SMBs and does not rehybridize with the cleaved aptamers. Given that the fluorescein of the cDNA-FAM is labeled at the 3′ terminus, the fluorescence signal intensity of cDNA-FAM is not significantly affected by the RecJ_f_ exonuclease digestion. The supernatant containing cDNA-FAM is collected via magnetic separation for subsequent fluorescence measurement.

### 3.2. Characterization and Sensing Properties of FAM-cDNA-Apt-SMBs

**Figure 1:**
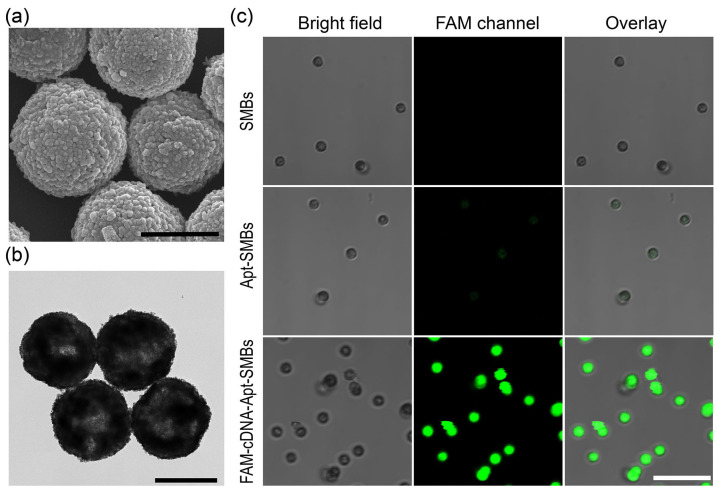
Characterization of the prepared FAM-cDNA-Apt-SMBs. (**a**) SEM and (**b**) TEM images of the FAM-cDNA-Apt-SMBs. Scale bar: 1 μm. (**c**) CLSM images of the SMBs, Apt-SMBs, and FAM-cDNA-Apt-SMBs. Scale bar: 10 μm.

In aptasensors, functional nucleic acids need to be immobilized at the solid-phase interface, so differences in immobilization level manufacturing lots as well as non-specific adsorption problems can directly affect the response performance of the entire sensing module or generate corresponding measurement errors. Herein, the aptamer/cDNA-FAM duplex was easily immobilized on commercial SMBs and effectively used as the target-recognition unit as well as the enzymatic substrate. Multiple repeated washes effectively reduced non-specific adsorption. The prepared FAM-cDNA-Apt-SMBs were characterized. SEM and TEM images were obtained to determine the morphology and size of magnetic beads (Figure 1a,b and Figure S1). The prepared FAM-cDNA-Apt-SMBs had a spherical shape with a rough surface, similar to the SMBs and the Apt-SMBs. Most of the beads were around 1 μm in diameter, with a few beads with diameters of about 2 μm (Figure S1). Fluorescence confocal measurements were utilized to verify the presence of aptamer/cDNA-FAM duplex on the FAM-cDNA-Apt-SMBs. As shown in Figure 1c, the green signal in the fluorescence confocal microscopy image reveals the distribution of the cDNA-FAM, indicating the successful self-assembly of cDNA-FAM on the surface of FAM-cDNA-Apt-SMBs. In contrast, the SMBs and Apt-SMBs show no signal in the FAM fluorescence channel. In addition, zeta potentials of the beads in buffer Ⅰ were recorded. The zeta potential of the SMBs is negative (−19.6 mV), probably because of the coating of streptavidin. As the aptamer and cDNA-FAM are gradually immobilized on the beads, the negative charges slightly increase step-by-step with the self-assembly of the fluorescent magnetic probe (Figure S2). Thus, the surface of the prepared FAM-cDNA-Apt-SMBs is also negatively charged (−23.3 mV), which would probably reduce the non-specific adsorption of negatively charged species such as proteins and nucleic acids. These observations suggested the successful preparation of FAM-cDNA-Apt-SMBs.

### 3.3. Feasibility of the Aptamer-Biosensing Assay

**Figure 2:**
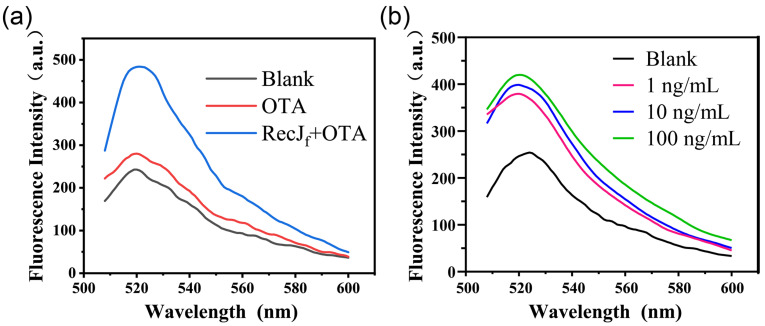
Feasibility analysis of the exonuclease-assisted aptasensor through fluorescence spectroscopy at 480 nm excitation wavelength. (**a**) The fluorescence emission spectra of the supernatant from blank, OTA, and OTA plus the RecJ_f_ exonuclease groups. (**b**) The fluorescence responses of the exonuclease-assisted aptasensor to different concentrations of OTA (0, 100 ng/mL, 1 μg/mL, and 10 μg/mL).

First, a native PAGE assay (12%) was performed to study the proposed aptamer-based competitive strategy (Figure S3a). The incubation of cDNA with the aptamer results in the appearance of a new band (lane 3), in addition to the bands of aptamer (lane 1) and cDNA (lane 2), owing to the hybridization of cDNA with the aptamer. The addition of OTA to the aptamer/cDNA duplex system results in a slight decrease in the brightness of the hybridization band (lane 4), which was attributed to the decomposition of the aptamer/cDNA duplex. Next, circular dichroism spectrometry was performed to analyze the conformation changes caused by the interaction of the aptamer/cDNA duplex with OTA. As shown in Figure S3b, the aptamer/cDNA duplex showed a positive characteristic peak at 280 nm and a negative characteristic peak at 252 nm. Theoretically, the positive peak at 280 nm is mainly formed by the stack of bases in the complementary region, while the negative peak at 252 nm is mainly caused by the right-handed helical structure [20]. With the addition of OTA, a significant decrease in the ellipticity of the positive peak is observed, indicating that the aptamer/cDNA duplex disintegrated because of the aptamer structure switch. To further verify the feasibility of the detection principle, reactions between 100 ng/mL OTA standard solution and the prepared FAM-cDNA-Apt-SMBs were performed in the absence or presence of the RecJ_f_ exonuclease. As shown in Figure 2a, the fluorescence emission spectra at 480 nm excitation wavelength exhibited a characteristic peak of fluorescein around 520 nm. Compared with the blank, the fluorescence signal of the supernatant was only slightly enhanced in the presence of 100 ng/mL OTA. Nevertheless, the addition of the RecJ_f_ exonuclease greatly enhanced the fluorescence signal (by almost two-fold compared to that without enzyme amplification), which is consistent with the exonuclease-assisted target recycling theory reported in the literature. Additionally, a dose-dependent fluorescence response for OTA is observed using this exonuclease-assisted aptasensor (Figure 2b). Therefore, the proposed aptamer-biosensing assay operated as intended.

### 3.4. Optimization of the Experimental Conditions

**Figure 3:**
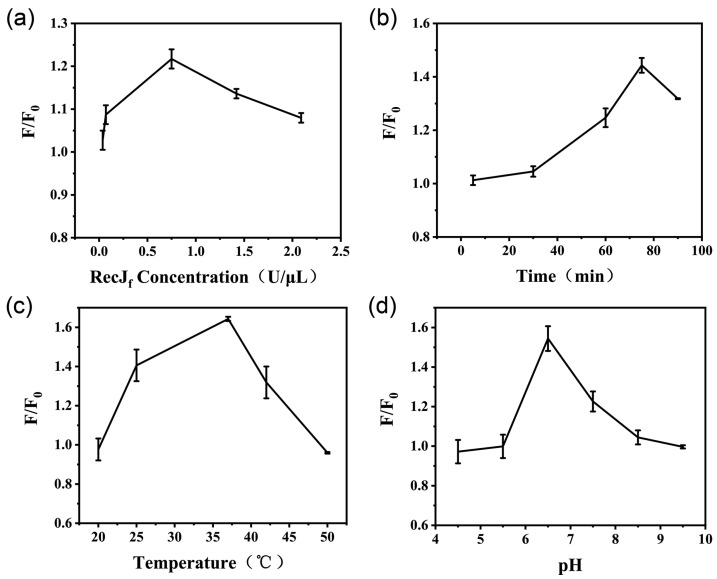
Optimization of the conditions of the exonuclease-assisted fluorescence assay. (**a**) Concentration of the RecJ_f_ exonuclease, (**b**) reaction time, (**c**) incubation temperature, and (**d**) pH level of the buffer were evaluated based on the fluorescent signal-to-noise ratio (*F/F*_0_), where *F* and *F*_0_ are the fluorescence intensities of the system in the presence and absence of OTA (5 ng/mL), respectively. The error bars represent the standard deviation of three parallel experiments.

To maximize the efficiency and sensitivity of the fluorescence assay, four crucial parameters, the concentration of the RecJ_f_ exonuclease, reaction time, incubation temperature, and pH of the buffer, were optimized. The optimization experiments were evaluated by the fluorescent signal-to-noise ratio (*F*/*F*_0_), where *F* and *F*_0_ are the fluorescence intensities of the system in the presence and absence of OTA (5 ng/mL), respectively. As the concentration of the RecJ_f_ exonuclease directly affects the efficiency of target recycling, the enzyme concentration was optimized first. As shown in Figure 3a, the *F*/*F*_0_ ratio increases with the concentration of the RecJ_f_ exonuclease, peaking at 0.75 U/μL. An excessive amount of the RecJ_f_ exonuclease can generate a non-specific background interference signal and cause unnecessary waste. Next, the reaction time of the assay was optimized by monitoring the *F*/*F*_0_ ratio at different time points. With increasing reaction time, the *F*/*F*_0_ ratio gradually increased and peaked within 75 min (Figure 3b). Thus, 75 min was chosen as the optimal reaction time. Moreover, the effect of incubation temperature on the fluorescence assay was evaluated, as shown in Figure 3c. The *F*/*F*_0_ ratio peaked at 37 °C. This can be explained by the poor activity of the RecJ_f_ exonuclease and the instability of the aptamer–cDNA duplex at lower or higher temperatures. Additionally, pH is another factor affecting the efficiency of the competitive reaction, as well as the enzymatic activity. As exhibited by Figure 3d, the highest signal-to-background ratio is observed at a pH of 6.5.

### 3.5. Sensitivity of the Proposed Method

**Figure 4:**
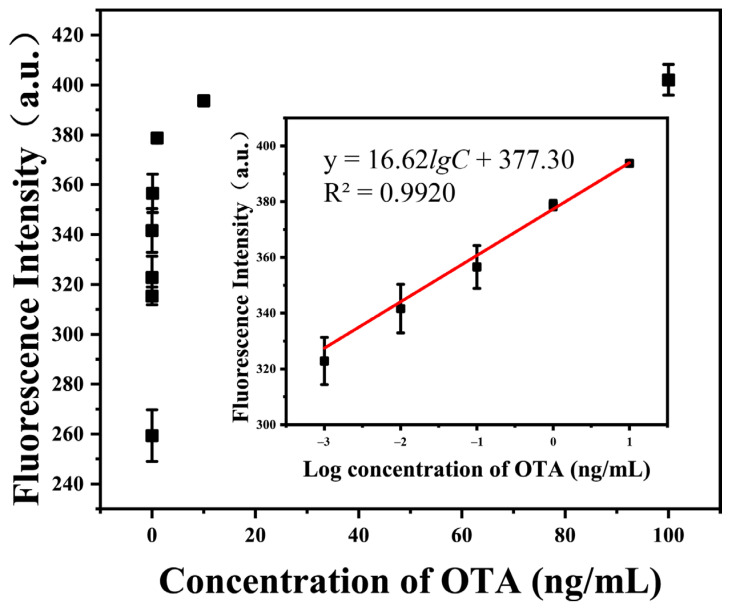
Fluorescent intensity signals of the proposed sensor at different concentrations of OTA (0–100 ng/mL) at 520 nm emission wavelength under optimized detection conditions. Inset: Corresponding linear calibration curve of the relationship between the fluorescence intensity at 520 nm and the logarithm of OTA concentration within the range from 1 pg/mL to 10 ng/mL. The error bars represent the standard deviation of three parallel experiments.

Under the optimized detection conditions, the fluorescence responses of the proposed sensor toward different concentrations of OTA were determined to study the sensitivity of the method. As shown in Figure 4, the fluorescence intensity of the system at 520 nm emission wavelength gradually increases with the concentration of OTA. A linear relationship (y=16.62lgx+377.30, $R^{2}=0.9920$) is observed between the fluorescence intensity and the logarithm of OTA concentration within the range from 1 pg/mL to 10 ng/mL (n=3). The limit of detection (LOD) was calculated as 0.31 pg/mL according to the 3σ rule. Combined with a modified pretreatment procedure described above, which dilutes the cereal sample at a ratio of 1/20 (g/mL), the proposed analytical method exhibited satisfactory performance in the quantitative analysis of OTA, with a wide linear range (1 pg/mL to 10 ng/mL) and high sensitivity (6.2 ng/kg of cereal) compared with other approaches, shown in Table 1 [6,24,25,26,27,28].

### 3.6. Sensitivity of the Proposed Method

**Figure 5:**
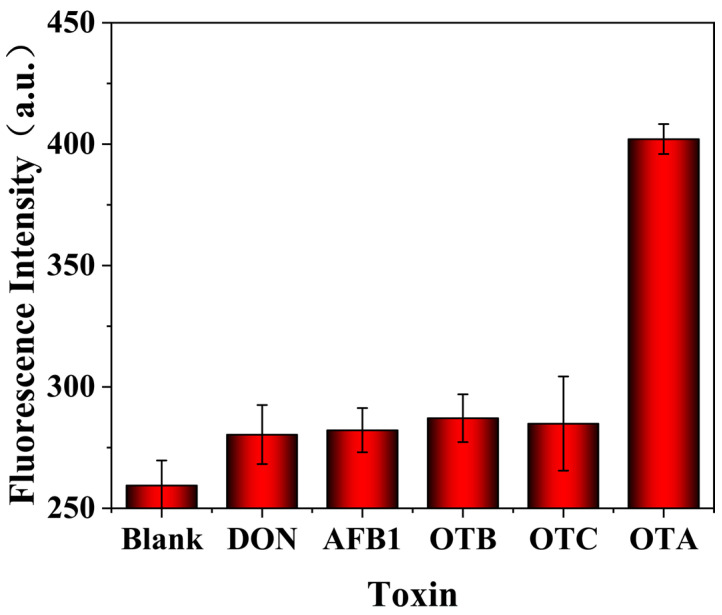
Fluorescent signals of the biosensing method toward different analytes: OTA (1 ng/mL), DON (500 ng/mL), AFB1 (500 ng/mL), OTB (500 ng/mL), and OTC (500 ng/mL). The error bars represent the standard deviation of three parallel experiments.

It is desirable to engineer a biosensing method that specifically responds to the target. Thus, the specificity of the proposed biosensing assay was studied by replacing OTA with its analogs (OTB and OTC), as well as other mycotoxins (DON and AFB1). As shown in Figure 5, the developed aptasensor exhibits a significant response to 1 ng/mL OTA, showing a considerably greater enhancement of fluorescence intensity compared to other mycotoxins (500 ng/mL, 500-fold the concentration of OTA). These results suggest that the proposed aptamer-biosensing method has good selectivity for OTA detection.

### 3.7. Determination of OTA in Real Samples

It is important to identify an aptasensor that can tolerate the interferences in complex matrices and work well in real samples. To validate the practical applicability of the proposed sensing system, cereal samples (wheat and corn flours) were spiked with different OTA doses (0.2, 2.0, and 20.0 μg/kg) and further extracted and diluted at a total ratio of 1/20 (g/mL), before analysis using the aptasensor. The performance of the aptasensor was compared with that of a conventional ELISA method (Table 2). By utilizing our aptasensor, the whole analytical method yields the recovery ranges of the spiked samples of 101.3–109.3% in wheat flour and 83.7–96.1% in corn flour, indicating a good tolerance to sample matrixes. All samples resulted in a repeatability relative standard deviation (RSDr, *n* = 3) of 8.0% maximum. Furthermore, the spiked concentration below 2 μg/kg of OTA was not detected by the ELISA method, which exceeded its detection limit. These results indicate that the proposed aptasensor exhibits better sensitivity and a larger detection range than ELISA. These results suggest that the aptasensor developed in this work has satisfactory feasibility and reliability for OTA detection in real cereal products.

## 4. Conclusions

We developed a novel OTA fluorescent sensor based on a facile fluorescent magnetic probe coupled with RecJ_f_ exonuclease-assisted target recycling amplification. Taking advantage of the biorecognition with the aid of aptamer sensing, our developed sensor exhibited a selective response to target molecules. Moreover, RecJ_f_ exonuclease cleavage was employed for the reuse of the target to improve the sensitivity of detection. Under the optimized experimental conditions, the proposed aptasensor enabled the ultrasensitive detection of OTA with a wide dynamic range and an LOD of 6.2 ng/kg of cereal. The experimental results also validate the feasibility and reliability of our method for OTA detection in real cereal products. Compared with the chromatographic method, which requires sophisticated instruments, and conventional immunoassay, which requires expensive antibodies, the developed method is cost-effective, simple in fabrication, and facile in operation. Compared with other aptasensors which use nucleic acid amplification to amplify signals, our aptasensor exploits target recycling to amplify specific signals that depend on target-aptamer recognition in each cycle, which avoids instability and non-specific amplification. Moreover, the provided strategy can be easily employed for detecting other targets of interest by replacing the aptamer, and thus has broad application prospects in food safety monitoring. Nevertheless, few aptasensors have reached the level of commercialization of other conventional sensors using enzymes and antibodies. Several challenges, including tolerance to environmental nucleases, resistance to fluctuations in conditions, and issues of immobilization and non-specific adsorption, remain to be overcome. More research is needed to apply the sensing strategy to other targets and to realize the full potential of the proposed aptasensor.

## Figures and Tables

**Table 1:** Comparison of the presented aptasensor with other published OTA detection approaches.

Sensor for OTA	Linear Range (ng/mL)	LOD (ng/kg)	Matrix
CdSe@CdS quantum dots, electrochemiluminescence aptasensor [24]	0.5–75	1780	Lily and rhubarb
Rolling circle amplification, DNA hydrogel sensor [25]	0.05–100	20	Beer
Cu_2_O@Fe(OH)_3_ nanocages, antibody-based immunosensor [26]	0.001–10	0.56	Millet and lake water
Hairpin-DNA-assisted electrochemical aptasensor [27]	0.03–10	13.3	Corn and wheat
DNAzyme amplified aptasensing platform, glucose meter [6]	0.001–300	0.88	Red wine
AuNSs (gold nanostars), AuNPs, SERS aptasensor [28]	0.005–0.25	20.6	Wine and coffee
RecJ_f_ exonuclease-assisted fluorescent aptasensor (this work)	0.001–10	6.2	Wheat and corn

**Table 2:** Application of the proposed aptasensor in the detection of OTA in cereal samples ^1^.

Samples	Added (μg/kg)	Our Method			ELISA Method		
		Detected (μg/kg)	Recovery (%)	RSD (%)	Detected (μg/kg)	Recovery (%)	RSD (%)
Wheat flour	20.0	21.21	106.0	8.0	20.91	104.6	5.1
	2.0	2.19	109.3	3.5	1.63	81.6	17.0
	0.2	0.20	101.3	2.4	Nd		
Corn flour	20.0	16.74	83.7	2.5	19.71	98.6	0.8
	2.0	1.92	96.1	6.8	1.97	99.0	10.3
	0.2	0.18	90.5	3.9	Nd		

^1^ *n* = 3, each result was obtained from three replications in parallel experiment. Nd: not detected.

## Data Availability

The data presented in this study are available on reasonable request from the corresponding author.

## Supplementary Materials

The following supporting information can be downloaded at: https://www.mdpi.com/article/10.3390/foods13040595/s1, Figure S1: SEM images of SMBs, Apt-SMBs and FAM-cDNA-Apt-SMBs. Figure S2: Zeta potential analyses of SMBs, Apt-SMBs, and FAM-cDNA-Apt-SMBs. Figure S3: Electrophoretic characterization and circular dichroism for feasibility verification.
