# Supplementary material for: Ultrasensitive Ochratoxin A Detection in Cereal Products Using a Fluorescent Aptasensor Based on RecJf Exonuclease-Assisted Target Recycling
Source: Foods. 2024 Feb 16;13(4):595. doi: 10.3390/foods13040595 (PMC10888426; doi:10.3390/foods13040595)
Supplement: Supplementary file 1 [file foods-13-00595-s001.zip › foods-2850907-supplementary.pdf]

Supplementary Material

Ultrasensitive Ochratoxin A Detection in Cereal Products Using a Fluorescent Aptasensor Based on RecJ$_f$ Exonuclease-Assisted Target Recycling

This file includes:

Figure S1. SEM images of SMBs, Apt-SMBs and FAM-cDNA-Apt-SMBs.

Figure S2. Zeta potential analyses of SMBs, Apt-SMBs and FAM-cDNA-Apt-SMBs.

Figure S3. Electrophoretic characterization and circular dichroism for feasibility verification.

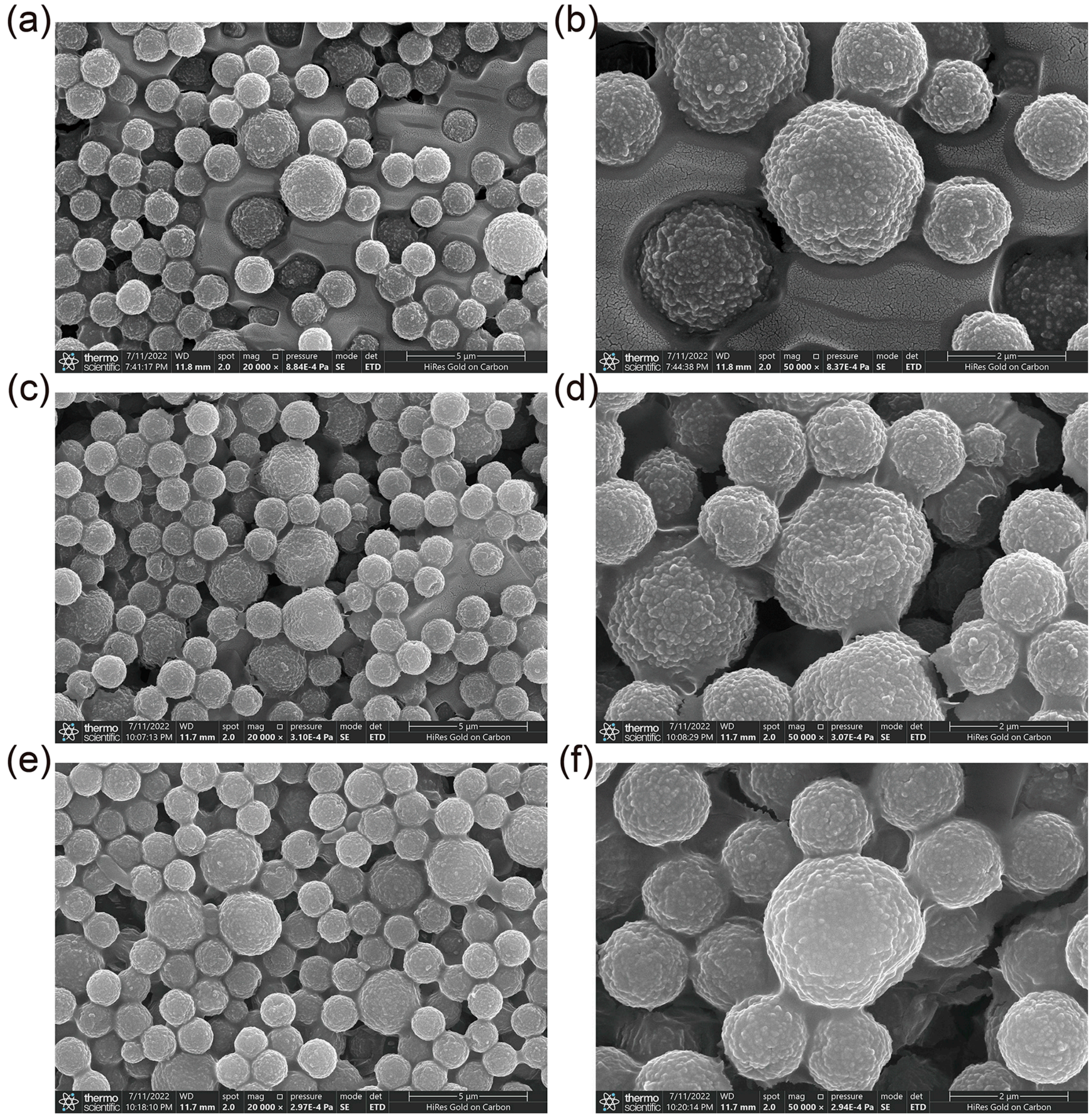

Figure S1. SEM images of SMBs (**a**, **b**), Apt-SMBs (**c**, **d**) and FAM-cDNA-Apt-SMBs (**e**, **f**). Scale bar: 5 μm (**a**, **c**, **e**) and 2 μm (**b**, **d**, **f**).

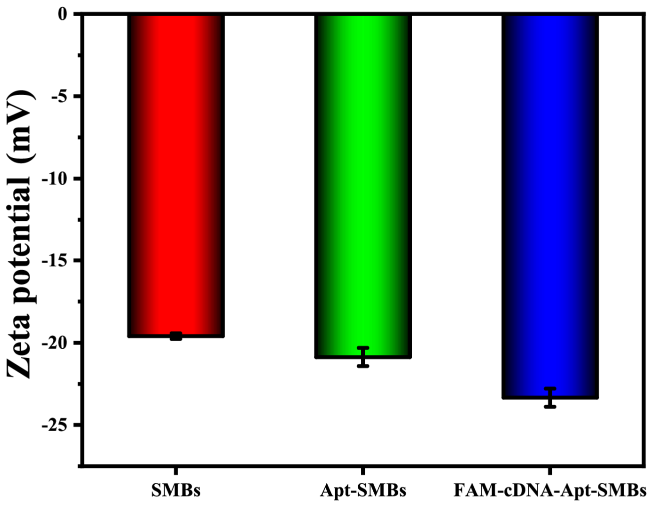

Figure S2. Zeta potential analyses of SMBs, Apt-SMBs and FAM-cDNA-Apt-SMBs.

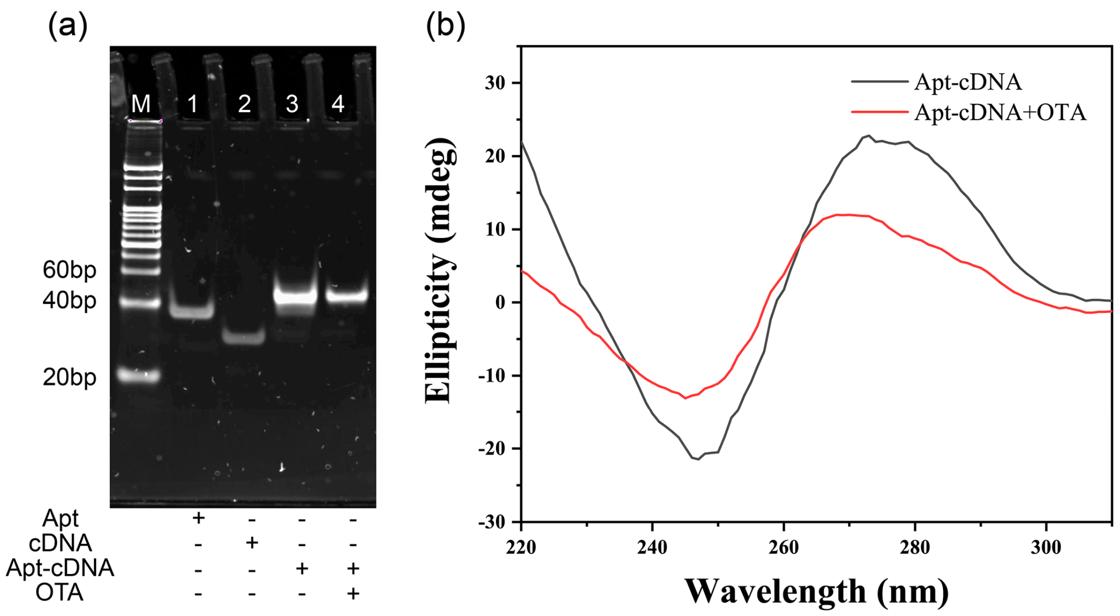

Figure S3. Electrophoretic characterization and circular dichroism analysis for feasibility verification. (**a**) The result of a native polyacrylamide gel electrophoresis (PAGE) assay (12%). Lane 1: Apt (1 μM); Lane 2: cDNA (1 μM); Lane 3: Apt-cDNA (1 μM); Lane 4: Apt-cDNA (1 μM) + OTA (1 μg/mL). The OTA aptamer/cDNA duplex were formed by hybridization at room temperature for 30 min (lane 3), while OTA were reacted with aptamer/cDNA duplex at 37 °C for 2 h (lane 4). (**b**) The circular dichroism analysis of aptamer/cDNA duplex (1 μM) and aptamer/cDNA duplex (1 μM) treated with OTA (1 μg/mL).
